# Leech-Like Parasites (Clitellata, Acanthobdellida) Infecting Native and Endemic Eastern Siberian Salmon Fishes

**DOI:** 10.1100/2012/652827

**Published:** 2012-05-02

**Authors:** Irina A. Kaygorodova, Elena V. Dzyuba, Nikolay M. Pronin

**Affiliations:** ^1^Limnological Institute of Siberian Branch of Russian Academy of Sciences, Ulan-Batorskaja Street 3, Irkutsk 664033, Russia; ^2^Institute of General and Experimental Biology of Siberian Branch of Russian Academy of Sciences, Sakhyanova Street 6, Ulan-Ude 670047, Russia

## Abstract

Salmonoid fish bdellosis is caused by leech-like ectoparasites in the monogenetic order Acanthobdellida. Although *Acanthobdella* species have been known to infect several threatened species in Eurasia, little is known about their ecology and epidemiology. In this paper, we report on the mass affection (up to 70.7%) of fish in lower course of the Chechuj River, a right tributary of the Lena and provide information on finding *Acanthobdella peledina* on two of six salmonoid fish species inhabiting there: lenok and grayling. New and more specific data on morphological peculiarities and feeding strategy were obtained. The ratio of body length to width in studied acanthobdellid collection is significantly less than one provided for the *A. livanowi* and the rest *A. peledina* from other water systems of Eurasia. Biology and lifestyle of the parasite population are revealed for the first time.

## 1. Introduction

The Acanthobdellida, a group of annelid worms, comprises of parasites of fish, which are restricted to the extreme northern parts of the northern hemisphere. The continued scientific interest in the Acanthobdellida is due to its mosaic combination of oligochaetous and hirudinean characters (leeches with setae), suggesting their intermediate role between Oligochaeta and Euhirudinea.

*Acanthobdella peledina* (Grube, 1851) is one of two known species of this group. Our basic knowledge of morphology of the *A. peledina* goes back to Livanow's monograph of 1905 [1] that was updated by the author in his publication in 1931 [2] in which this species was identified as an ancient hirudinean and close phylogenetic relationships between both the Hirudinea as well as the Oligochaeta were proposed [2, 3]. A series of investigations has corroborated Livanow's phylogenetic hypothesis. The “living relic” *A. peledina* in fact occupies a transitional phylogenetic position between the oligochaetes and the leeches, as shown by morphological and life cycle investigations [4–8] and molecular phylogenetic analyses [9–11]. 

These archaic freshwater leech-like clitellates belonging to Acanthobdellida are semipermanent parasites, restricted almost exclusively to salmonoid fish. In contrast to its congener *A. livanowi* (Epstein, 1966), which has a limited range in fresh waters of Kamchatka and Chukotka Peninsulas and a poorly studied biology [12], *A. peledina* has a wide range of habitat at high latitudes of the Northern Palearctic—from Norway on the west to the Kolyma Region on the east [13] and in North America [14, 15]. *Acanthobdella peledina* is psychrophilic and stenoecic species inhabiting oligotrophic waters exclusively. Its area of distribution is much smaller compared with that of their hosts—Salmonidae and Thymallidae [16–18]. Ecologically, *A. peledina* is more stenoecic than fishes, which it infests. Since acanthobdellids have a relatively low frequency of occurrence, it is obvious that their total number in distinct populations of the Siberian reservoirs is very low. Therefore, despite a wide area, the acanthobdellids are classified as rare species and listed as endangered in Buryatia and the Irkutsk Region.

In the second half of the 20th century, the abundance and habitat area of *A. peledina* declined including in lakes Onega and Ladoga due to a reduction in its obligate hosts and eutrophication in reservoirs [19]. The importance of maintenance of this “living fossil” is difficult to overestimate. In this regard, each new finding on *A. peledina* is important both for understanding poorly studied aspects of its life cycle and ecology, and for studying its role in the evolution of segmented worms as a whole.

## 2. Material and Methods

### 2.1. Geographical Description of Sampling Location

**Figure 1:**
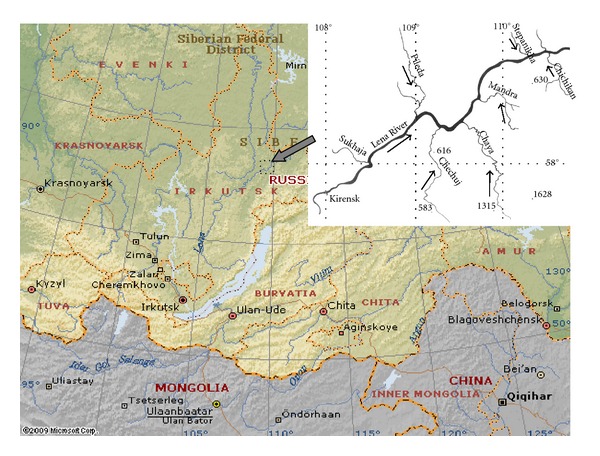
Location of the study region (left) and schematic map of the sampling area (right).

The Chechuj River is a right tributary of the Lena River (Figure 1). The Chechuj is one of the most beautiful rivers in the Irkutsk Region, it comes out from a small crystal clear lake situated at an altitude of 1020 m, in the mountain site where two ridges the Atikansky and the Chuysky converge. This river is wild and almost inaccessible during winter and flood time. Every year huge numbers of different fish species come here for feeding and spawning.

### 2.2. Ichthyologic Analysis

We used materials collected by Dr. Elena Dzyuba in August of 2009 and 2010 from the Chechuj River (Figure 1). Since the river is a specially protected natural area, access to this river is strictly forbidden in periods of spawning of valuable fish species (grayling and lenok in April–June and all whitefishes in September-October). Fishes were caught in 6 km from the mouth of the river by gill nets (32–40 mm mesh) with a total length of 100 m. The primary and laboratory processing of the fish material was carried out by conventional methods [20, 21]. The number and size-weight characteristics of the studied species are presented partially in Tables 1 and 2, and in Section 3.

### 2.3. Morphological Analysis of Parasites

Leech-like clitellates were collected by hand directly from infected fish and were fixed in 80% ethanol. Initially, Dr. E. Dzyuba sent specimens to Dr. Nikolay Pronin who did species determination according to the original description [1] and previous identification keys [13].

Morphological analysis has been conducted by Dr. Irina Kaygorodova using a stereomicroscope WILD M4C-61 149 and a binocular microscope Axiostar plus (Carl Zeiss Microimaging Gmb). Photos of the parasite were taken with a camera NIKON D700.

## 3. Results

Parasitological inspections of fish in the lower course of the Chechuj River (Figure 1), a right tributary of the Lena River (N 58°09′, E 109° 14.5′) were conducted in 2009 and 2010. Two hundred seventy-four individuals of salmonoid fishes in 2009 and four hundred forty-eight individuals of salmonoid fishes in 2010 were examined (Table 1). Whereas in 2009 the prevalence was not more than 5.6%, in 2010 the larger parasitemia was recorded (Table 1). Acanthobdellids were found on two salmon species—a local grayling and the lenok. They were not reported on other four salmon species inhabiting the river, including the taimen *Hucho taimen*, whitefishes (*Coregonus pidschian* and *Coregonus tugun*), and the round whitefish *Prosopium cylindraceum*.

Two out of 45 lenoks in 2009 and on 14 lenoks out of the total catch in 2010 suffered from the bdellosis. Only 11 infected individuals from 195 studied graylings were reported in 2009. The maximal infestation was observed in the Chechuj River graylings in 2010—195 of 276 captured individuals bore acanthobdellids on the body. The total prevalence ranged from 4.4 to 70.7% (Table 1).

**Figure 2:**
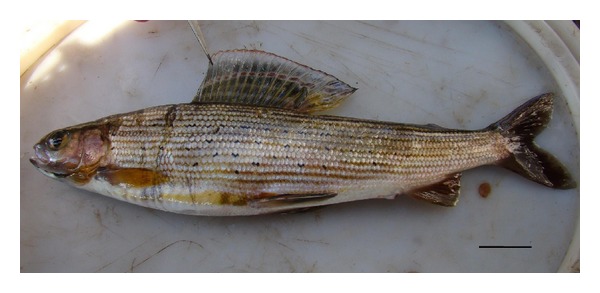
Localization of the acanthobdella parasite on the dorsal fin of Lena grayling (*T. arcticus baicalensis*). Bar is equal to 2.5 cm.

**Figure 3:**
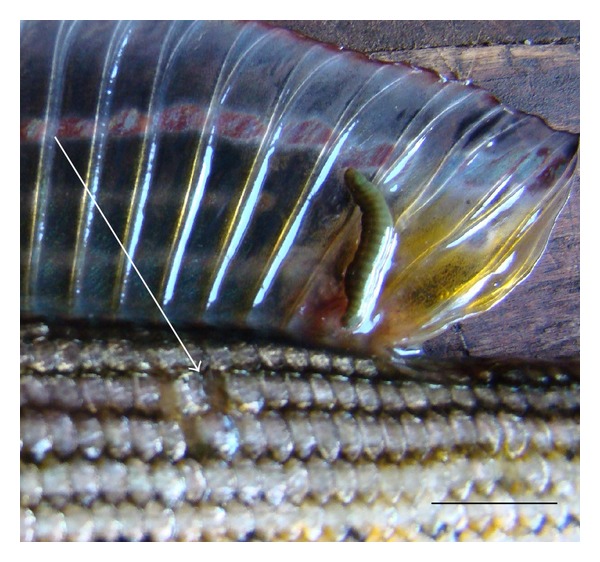
Exterior view of a living *Acanthobdella peledina* from the Chechuj River. The arrow points to three ulcerous openings left by the parasite in the soft tissues of their host. Bar is equal to 1 cm.

The infection intensity was from 3 to 8 specimens of parasites per one fish with a modal interval of this index 5-6. Parasites were situated at fins, in preference at the base of the dorsal fins, forming a cluster of deep ulcerous openings in soft tissues of their host (Figures 2 and 3).

### 3.1. Taxonomic Review and a Brief Description of Species Affected by the A. Peledina

Acanthobdellids were found on two species of salmon fishes: *Thymallus arcticus baicalolenensis *Matveev et al., 2005 (Figure 2) and *Brachymystax lenok* (Pallas, 1773). Infected fishes belong to two different families of Salmoniformes: Thymallidae (Gill, 1884) and Salmonidae (Cuvier, 1816). The classification of higher rank taxa is given according to Bogutskaya and Naseka [22]. 

#### 3.1.1. Salmoniformes: Thymallidae

*Thymallus arcticus baicalolenensis* Matveev et al., 2005.

**Distribution**

Lena River Basin, upper streams of northern tributaries of Lake Baikal (Tyya River, Upper Angara River, Barguzin River), upper streams of Left Bureya, Uda Rivers.

**Species Status**

Object of amateur fishing.

**Age-Size Characteristics**

Maximum age is 9+ years, maturing in 2–5 years, maximum length of 37.3 cm, weight of 0.77 kg [23]. Parasitologically studied Lena graylings from the Chechuj River were 12.0–36.3 cm in length and with a weight of 0.03–0.36 kg. Only a young age group of mature fishes suffered from acanthobdellosis: four two-year-old individuals of 2009 and 78 individuals of 2010; five three-year-old individuals of 2009 and 71 individuals of 2010; two four-year-old individuals of 2009 and 46 individuals of 2010. According to modal number, preferably two years individuals of both sexes had suffered from bdellosis. Information on age structure, size-weight characteristics, and sex ratio of infected fishes are provided in Table 2.

#### 3.1.2. Salmoniformes: Salmonidae

*Brachymystax lenok *(Pallas, 1773).

**Distribution**

Palearctic: Siberian species. It inhabits the rivers of Siberia from the Ob River to the Kolyma River. It occurs in the Amur River basin, the rivers flowing into the Sea of Okhotsk, and the Sea of Japan. It is widespread in the basins of the upper reaches of the Lena and the Kirenga River, but mainly in the tributaries of mountainous type.

**Species Status**

Not numerous species. Object of amateur fishing.

**Age-Size Characteristics**

Size and weight of the lenok vary considerably depending on the area of habitation. Maximum age of lenok in the Lena River is 18+ years, with an average body length of 65.5 cm and weight of 2.4 kg. In our catches from the Chechuj River, mainly mature individuals 18.2–51.2 cm in length and weighing 0.56–1.4 kg were met. All infected individuals belong to young mature age group: one four-year-old individual of 2009 and two individuals of 2010; four five-year-old individuals of 2010; one six-year-old individuals of 2009 and five individuals of 2010; three seven-year-old individuals of 2010. Five-year-old males and seven-year-old females prevail among infected individuals. Measurements of infected fishes as well as their age and sex ratio are presented in Table 2.

### 3.2. Morphological Features of A. Peledina from the Chechuj River

20 individuals fixed with 80% alcohol solution were available for morphological analysis. Below there is a brief description of external morphological characters of examined acanthodbella.

**Body Dimensions**

Taken from different hosts, the specimens of *A. peledina* have similar body proportions. The length of the worms ranges from 15 to 22 mm and 3–7 mm in width. The largest specimen of 22 mm in length is 6 mm in width. The maximum width of 7 mm was recorded in a specimen of 20 mm in length. The average body length of the Chechuj acanthobdellids is 18.4 mm with 4.65 mm in width (Table 3).

**Figure 4:**
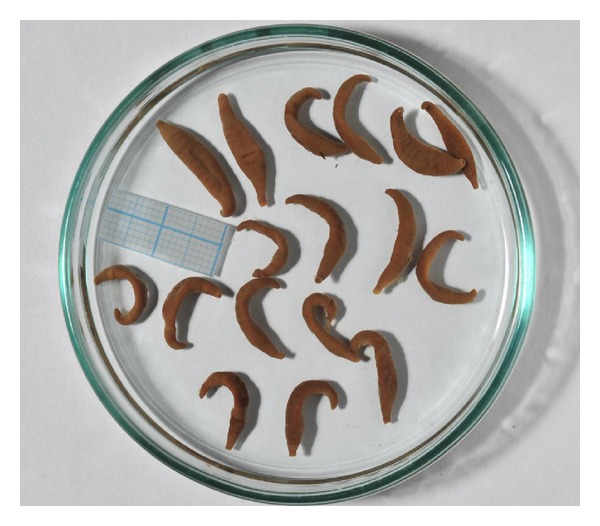
Exterior view and size variation of ethanol-fixed acanthobdellids from the Chechuj River.

*Body shape* of studied individuals varies slightly. Living specimens had vermiform shape (Figure 3), whereas all ethanol fixed specimens are smooth spindle-shaped form, slightly oblate dorsoventrally (Figure 4). The ratio of body length to width in the Chechuj acanthobdellid collection ranged from 2.83 to 6, with an average of 4.14 (Table 3). This parameter is significantly less than one provided for both the *A. livanowi* (Epstein, 1966), living in the Kamchatka and Chukchi Peninsulas, and the rest *A. peledina* of inland water reservoirs and watercourses of Eurasia (Table 3).

*Colour* of living parasites was uniform from green to brownish green (Figure 3). Only the anterior end of the body was distinguished by yellowish coloration. Fixed samples quickly lost native marking and became uniform light-brown clay (Figure 4).

**Eyes**

Three pairs of dark-red eyes, which are well distinguishable in the living individuals and almost invisible because of the rapid bleaching of the pigment in fixed specimens.

**Anterior End of the Body**

**Figure 5:**
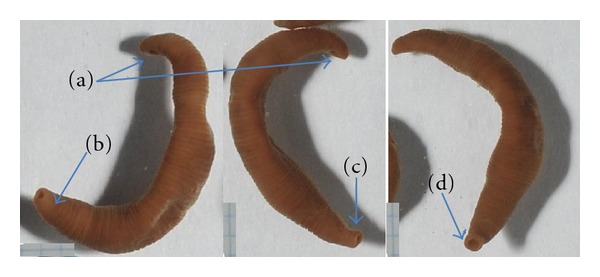
Morphology of the A. peledina from the Chechuj River: (a) anterior sucker, (b) posterior sucker; (c) elongated posterior end of the body; (d) posterior sucker in a normal state.

**Figure 6:**
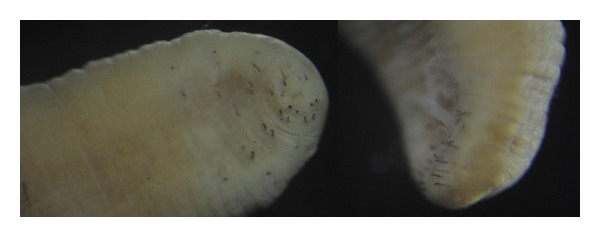
Dark, sharply declinate seta on anterior end of body of the *A. peledina* from the Chechuj River.

The anterior sucker is not developed. The head portion includes the first five segments. It is not separated from the adjacent body part in contrast to *A. livanowi*, which has an obviously separated head and more or less developed anterior calyciform sucker. The dorsal surface of the head is convex, ventral one is flat or concave slightly (Figure 5). The mouth pore is a small opening on the forward portion of the first segment (Figure 6).

**Setae**

In all examined* A. peledina*, there are 40 setae, which are located only on the first ten annuli of five segments of the anterior part of the body (Figure 6) that according to Dahm [24] has species-specific pattern. Each segment has 8 of setae arranged in transverse rows, in each row setae are grouped into four bundles. Each bundle contains two setae. A similar structure of seta apparatus (4 bundles of 2 setae) is present in representatives of the family Lumbriculidae (Annelida, Oligochaeta). In contrast to lumbriculids all setae of *A. peledina* are dark brown, hook-shaped, and their location is restricted to the anterior sucker.

**Posterior End of the Body**

It is lengthened or retracted inward (Figure 5). Small-sized sucker is rounded (Figures 4 and 5).

## 4. Discussion

### 4.1. List of Fish Hosts

While traveling through Siberia in 1842–1845, Alexander Middendorf, a Russian naturalist, discovered, unknown at that time, parasitic worms on the peled (*Coregonus peled*) in lower reaches of the Yenisei River. He gave them to his colleague, Swiss zoologist E. Grube who described the species, and referred it to a new genus *Acanthobdella* (Grube, 1851). Later it became clear that this leech is a specific parasite of salmonoids [1–3, 25] and many other. 

Previously, 18 fish species related to 8 genera were identified as the hosts of the *A. peledina* [16]. According to current knowledge, the list of fish-host species for *A. peledina* includes 25 species related to 10 genera (Table 4). The fishes found to be parasitized by *A. peledina* belong to the families Salmonidae, Coregonidae, Thymallidae, Lotidae, and Bothidae. Representatives of the latter two families are very doubtful. The burbot *Lota lota* was only once mentioned as a host of *A. peledina* in Lake Imandra [26], then this information was spread by multiple citations. Regard to the Bothidae, information about *Scophthalmus maximus* parasitized by *A. peledina*, we found only in Hauck et al. [15], where it was referenced without specifying the place of capture. Anyway, a checklist of host species of these clitellate parasites was enlarged from 18 to 25. Main of them belongs to three families Salmonidae, Coregonidae, and Thymallidae (Table 3).

### 4.2. Diet of the Leeches—Blood or Epidermis

In spite of appearance of some publications [27–30], feeding strategies of the majority of leech species remain unknown and studied insufficiently. In this situation the ancient leech *A. peledina* is not an exception. Dahm [24], when making a laboratory experiment, observed the behavior of *A. peledina* attached to a salmonoid fish and mentioned that after the death of the fish, leeches continued to feed on their host about one hour. We observed the comparable situation for worms in nature. Acanthobdellae did not left immediately dead fish. They were attached tight onto a fish during 1.5–2 hours after fish death. 

During the study we noted that *Acanthobdella* may occur anywhere on the body of its hosts, but usually the leeches attach to the base of the dorsal fin by the posterior sucker (Figure 3). It was affirmed that after attaching the setae of the head region to the skin of a fish, the ectoparasite feeds on blood and tissue of its vertebrate host [30]. There are numerous rhynchobdellid leeches living attached to the skin of fish, and unless these parasites have an effective means of penetrating the dermis and reaching the blood capillaries, it seems that they are also epidermis feeders. Nevertheless the reports of the occurrence of albuminous fluid with no resemblance to blood in the oesophageal diverticula of some species [31], are more consistent with a diet of epidermis than with one of blood. Our observations support the hypothesis of epidermis feeding than bloodsucking. *Acanthobdella* individuals produce a round wounds on the skin of salmonoid fishes (Figure 3) which could serve as a basis for bacterial or fungal infections, increase stress and lead to fish kills.

### 4.3. Life Cycle

The life cycle of *A. peledina *was studied by Dahm [24] and Andersson [32] who kept leeches in aquaria. The information they obtained can be summarized as follows. Copulation occurred more often at 4°C than at the higher temperature during August/September. In one week after copulation, the free-living individuals produce cocoons. Thereafter, the soft translucent cocoon is attached to the substrate and later becomes hard and dark brown. Free-living young leeches can be observed over a period of several months. The process is reminiscent to that observed in oligochaete worms considered to be the original state in leech [8, 27]. The first infested fishes were found next year by the end of September. Young *A. peledina *remain attached to their host over the subsequent year and reach maturity. Then, the adult individuals leave their host, produce cocoons, and die a few months later.

**Figure 7:**
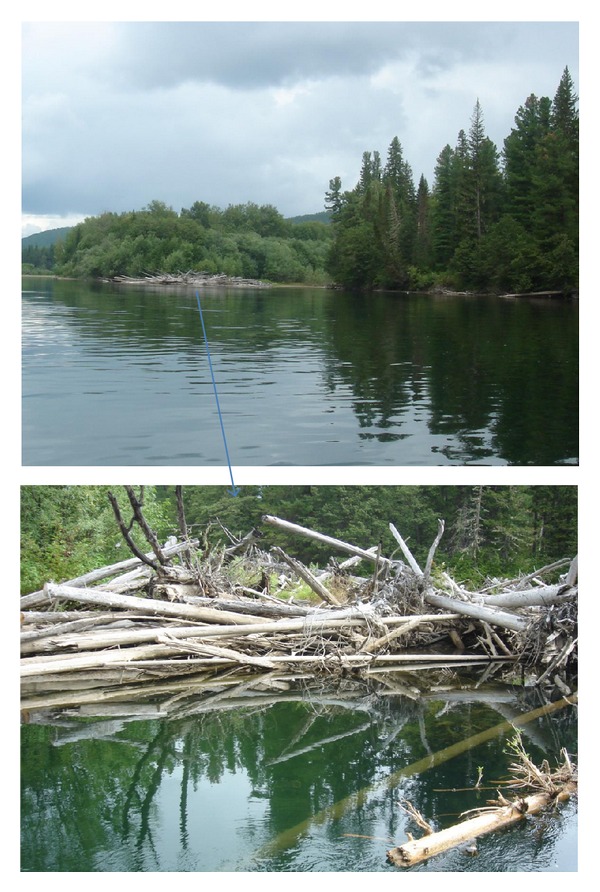
Numerous subsurface “log gorges” on the river, a habitat of acanthobdellids.

According to our data, the appearance of these parasites on fish in the Chechuj River recorded from the second half of August. By the end of September, worms reach sexual maturity and weight of over 200 mg after that they leave the victim. In another season these leeches were not found [16, 17]. Our observations are supported by data from surveys of local fishermen. The subsequent fate of the parasite is unknown. Unfortunately, *A. peledina* was never found at free-living stage in Siberia. Most likely, the worms reproduce and develop in the coastal zone in the upper reaches of the river, in the so-called “log gorges” (Figure 7) formed by natural accumulation of fallen trees. Indeed submerged rotten wood is a favorite habitat for different leeches. This suggests that the infection of fish is most probable in these places, when fishes are easy to access.

As stated above, leech parasites were found only on two of five salmonoid species inhabiting the Chechuj River. During the summer, both the lenok and the grayling stayed mainly in the tributaries. By winter they descend into the main channel of the Lena River. Spawning migration in tributaries begins in mid-May at water temperatures of 6–8°C, when juveniles and spawners migrate upstream for feeding and reproduction. “Seaward” run ends in early October. Exactly during this period, acanthobdellosis was registered on fishes of the Chechuj River. Moreover, all interviewed local fishermen reported that only shoals of the lenok and the grayling during summer months stay in the numerous “log gorges.” Thus, the life cycles of *Brachymystax lenok*,* Thymallus arcticus baicalolenensis*, and *Acanthobdella peledina* coincide.

## Figures and Tables

**Table 1:** Infection of Chechuj River fishes with parasitic worms of the genus *Acanthobdella*.

Year		*B. lenok*	*T. arcticus baicalolenensis*	*P. cylindraceum*	*C. pidschian*	*C. tugun*	*H. taimen*
2009	N	45	195	8	26	—	—
	P.	4.4	5.6	—	—	—	—

2010	N	78	276	65	24	4	1
	P.	18.0	70.7	—	—	—	—

N: Number individuals; P.: prevalence in %.

**Table 2:** The size-weight characteristics of infected fishes in 2009 and 2010.

Year	Species	Age	Body Length, cm		Weight, kg		Sex ratio (M/F)
			Average (std div.)		Average (std div.)		
			Actual range		Actual range		
			Male	Female	Male	Female	
2009	Lenok	4+	32.7	—	0.345	—	1/0
		6+	—	49.9	—	1.20	0/1
	Grayling	2+	20.1 (1.70)	15.8 (1.06)	0.07 (0.02)	0.03 (0.01)	1/1
			18.9–21.3	15.0–16.5	0.06–0.08	0.03–0.04	
		3+	24.0 (0.85)	24.1 (0.14)	0.12 (0.02)	0.11 (0.01)	3/2
			23.0–24.6	24.0–24.2	0.11–0.14	0.10–0.12	
		4+	26.5	25.5	0.16	0.14	111

2010	Lenok	4+	34.1 (2.62)	—	0.40 (0.10)	—	2/0
			32.2–35.9		0.33–0.47		
		5+	41.2 (1.16)	—	0.77 (0.05)	—	4/0
			41.0–42.9		0.72–0.84		
		6+	49.2 (0.90)	49.9	1.20 (0.04)	1.20 (0.01)	3/2
			48.2–49.8		1.14–1.21	1.19–1.21	
		7+	—	50.8 (0.46)	—	1.36 (0.09)	0/7
				49.9–51.2		1.19–1.43	
	Grayling	2+	20.6 (2.65)	19.8 (3.0)	0.08 (0.03)	0.07 (0.03)	42/36
			15.9–24.5	15.0–24.5	0.04–0.14	0.03–0.14	
		3+	24.0 (1.89)	23.7 (1.21)	0.12 (0.03)	0.12 (0.02)	36/35
			19.4–26.7	22.2–26.0	0.06–0.16	0.09–0.16	
		4+	26.2 (0.86)	25.0 (1.28)	0.16 (0.01)	0.14 ± 0.02	16/30
			24.5–27.5	23.5–27.1	0.14–0.18	0.12–0.18	

**Table 3:** The body ratios of specimens from the Chechuj River and similar data by Epstein.

Source of data	Species	Body length (L), MM	Maximal body width (W), MM	L/W	Number of examined individuals
		Average (std dev.)	Average (std dev.)	Average (std dev.)	
		Actual range	Actual range	Actual range	
Our data	*A. peledina*	18.4 (1.85)	4.65 (1.09)	4.14 (0.88)	20
		15–22	3–6	2.83–6.00	
Data by Epstein [12]	*A. peledina*	?	—	5.7	23
		19–32		4–8	
	*A. livanowi*	?	—	4.5	14
		2.5–14		2.7–8	

**Table 4:** List of fish species and subspecies, which are parasitized by *A. peledina. *

Number	Species	Locality (reference)
Salmonidae		
1	*Brachymystax lenok* (Pallas, 1776)	Lena River Basin [16]
2	*Hucho taimen* (Pallas, 1773)	Lena River Basin [16]
3	*Salvelinus alpinus* (Linnaeus, 1758)	Lake Onega [33, 34] Kara River Basin: Lake Nyarmata [35]
4	*Salvelinus erythrinus* Georgi, 1775	Basins of Lake Baikal and Lena River [16]
5	*Salvelinus lepechini* (Gmelin, 1789)	Lake Onega [33]
6	*Salvelinus neiva *(Taranetz, 1933)	Far east: Okhota River [36]
7	*Salmo trutta* (Linnaeus, 1758)	Norwegian freshwaters [37] Lake Pyaozero [38]
8	*Salmo salar* (Linnaeus, 1758)	Lake Onega, Kamennaya River [38]

Coregonidae		
9	*Coregonus tugun *(Pallas, 1814)	? [15]
10	*Coregonus autumnalis* (Pallas, 1776)	Ob River Basin [25, 39]
11	*Coregonus lavaretus lavaretus* (Linnaeus, 1758)	Kola Peninsula: Lake Imandra [26]
12	*Coregonus muksun* (Pallas, 1814)	Enisej River [25]
13	*Coregonus nasus* (Pallas, 1776)	Enisej River; Anadyr River [25]
14	*Coregonus peled *(Gmelin, 1789)	Enisej River [40]
15	*Coregonus pidschian *(Gmelin, 1789)	Lena River, Upper Angara River [17]
16	*Coregonus sardinella* (Valenciennes,1848)	? [13] Alaska: Chipp River [15]
17	*Prosopium cylindraceum* (Pallas, 1784)	? [13]
18	*Stenodus leucichthys *(Gueldenstaedt, 1772)	Enisej River Basin [25]

Thymallidae		
19	*Thymallus arcticus* (Pallas, 1776)	Pechora River Basin [13]
20	*Thymallus arcticus baicalensis* (Matveev et al., 2005)	Lena River Basin [16]
21	*Thymallus baicalensis *(Dybowski, 1874)	Upper Angara River [17]
22	*Thymallus pallasii* (Valenciennes, 1848)	Lena River Basin [16]
23	*Thymallus thymallus* (Linnaeus, 1758)	Kola Peninsula: Lake Imandra [41]

Lotidae		
24	*Lota lota* (Linnaeus, 1758)	Kola Peninsula: Lake Imandra [26]

Bothidae		
25	*Scophthalmus maximus *(Linnaeus, 1758)	? [15]
